# Beneficial Effects of *Lactobacillus delbrueckii subsp. lactis* N102 and *Lactobacillus sakei* H1-5 Added as Starter Strains on the Metabolome, Safety and Quality of Dry-Fermented Sausages

**DOI:** 10.3390/foods14101675

**Published:** 2025-05-09

**Authors:** Yushan Jiao, Min Cai, Wensheng Tang, Zhengkai Wang, Yingli Liu

**Affiliations:** Key Laboratory of Geriatric Nutrition and Health, Ministry of Education, Beijing Technology and Business University (BTBU), 11 Fucheng Road, Beijing 100048, China; jiaoyushan0601@163.com (Y.J.); caimin0901@163.com (M.C.); qttws1012@163.com (W.T.); 18731023358@139.com (Z.W.)

**Keywords:** lactic acid bacteria, dry-fermented sausage, nitrite, biogenic amine, edible quality, non-volatile metabolites

## Abstract

This study investigated the beneficial effects of individual and co-inoculation with *Lactobacillus delbrueckii subsp. lactis* N102 and *Lactobacillus sakei* H1-5 on improving safety parameters, sensory characteristics, and non-volatile metabolite profiles in dry-fermented sausages. Comprehensive analyses were conducted throughout the 20-day maturation period (0, 6, 13, 16, and 20 days), including physicochemical monitoring (moisture content, malondialdehyde (MDA) levels, biogenic amine concentrations, and sodium nitrite residues); sensory evaluation (color parameters and textural properties); and ^1^H NMR-based metabolomic profiling. Key findings revealed strain-specific advantages: the N102 inoculation significantly delayed lipid oxidation, achieving the lowest final MDA concentration (4.5 mg/kg) among all groups. Meanwhile, H1-5 supplementation notably improved color attributes (a*/b* ratio = 1.34). The co-inoculation strategy demonstrated synergistic effects through (1) accelerated acidification (pH 5.3 by day 6); (2) enhanced textural properties (significantly increased hardness and elasticity vs. control); (3) optimized water distribution (free water reduced to 0.56% with 64.73% immobilized water); and (4) a significant reduction in sodium nitrite residues (70% decrease) and complete elimination of phenylethylamine (total biogenic amines: 702.94 mg/kg). ^1^H NMR metabolomics identified 30 non-volatile metabolites, and the co-inoculation significantly increased the amount of essential amino acids (leucine, isoleucine), flavor-related compounds (glutamic acid, succinic acid), and bioactive substances (gooseberry, creatine). These metabolites enhanced antioxidant capacity, freshness, and nutritional value. Our findings demonstrate that strategic co-cultivation of food-grade lactobacilli can synergistically enhance both the techno-functional properties and biochemical composition of fermented meat products, providing a viable approach for quality optimization in industrial applications.

## 1. Introduction

In Europe, fermented meat products constitute 20–40% of total processed meat production, primarily in the form of fermented sausages. These products are a key component of meat consumption, accounting for 3–5% of the total meat intake across the region. Significant diversity exists among countries: Spain offers over 50 types of fermented sausages, while Germany boasts more than 350 varieties. Additionally, Germany, Spain, France, and Italy collectively consume over 600 million kilograms of fermented sausages annually, highlighting both their rich typological diversity and substantial market presence in European dietary culture [1]. Dry-fermented sausage is a product prepared by grinding lean pork together with fat, followed by mixing with a variety of spices and stuffing into casings, which is then subjected to continuous fermentation and drying in a drying chamber [2]. During the fermentation process, the microorganisms from the raw materials and the environment play a pivotal role in shaping the flavor profile [3]. Consequently, starter cultures are commonly added to the sausage to control the presence of microorganisms that alter the meat texture, ensuring stability and safety, as well as enhancing the sensory attributes of the final product [4]. Most microorganisms thrive at high water activity (Aw) values. In dry-fermented sausages, microbial activity is regulated by pH (4.5–5.5) and Aw (0.85–0.98). Lactic acid bacteria (LAB) inhibit pathogen proliferation via rapid acidification (pH < 5.0) during the initial stage, while acid-tolerant coagulase-negative staphylococci (CNS) and yeasts secrete lipases and proteases during maturation, catalyzing the formation of free fatty acids and flavor peptides. Additionally, lipid oxidation during maturation represents one of the primary biochemical determinants of product quality, linked to changes in flavor, color, texture, and mouthfeel [5,6]. Moderate oxidation generates volatile compounds such as aldehydes and ketones, contributing characteristic aromas like nutty and smoky notes. Conversely, excessive oxidation leads to rancidity (e.g., malondialdehyde formation), color deterioration, and nutrient loss, necessitating strategies such as antioxidant addition or microbial inhibition to mitigate over-oxidation.

Starter cultures are products containing viable or dormant microorganisms that exhibit desired metabolic activities in fermentation matrices [7]. Commercial fermenting agents currently consist primarily of *lactic acid bacteria* (LAB), *coagulase-negative staphylococci* (CNS), yeasts, and fungi, with each microbial group contributing to unique product characteristics through synergistic interactions. As core fermenting species, LAB achieve rapid acidification of the matrix by metabolizing carbohydrates to produce lactic acid. This process not only effectively inhibits pathogenic growth but also drives proteins toward their isoelectric point, reducing electrostatic repulsion and enhancing bonding capacity, thereby cross-linking fibrin molecules into a dense network that traps moisture and components, improving firmness and cohesiveness. Additionally, LAB degrade proteins and peptides to generate free amino acids, serving as precursors for various flavor compounds [8]. Chen et al. [9] have demonstrated that inoculated sausages exhibit lower levels of volatile compounds associated with lipid oxidation (e.g., aldehydes, ketones, hydrocarbons), which helps suppress the formation of unpleasant flavors. The inhibition of pathogenic and spoilage bacteria by LAB species is primarily attributed to the production of organic acids or other antimicrobial metabolites, such as hydrogen peroxide, diacetyl, and peptides termed bacteriocins [10]. Beyond LAB, CNS also contribute to the development of typical flavors and colors in fermented meat products. Studies have shown that certain staphylococci in fermented sausages possess lipase and protease activities linked to flavor development. Yeasts and fungi, serving as adjunct starters in sausage fermentation, play critical roles in achieving multifaceted quality attributes, including antimicrobial activity, antioxidation, nitrite degradation, and pathogen prevention [11] *Debaryomyces hansenii*, the most common yeast in sausages, can buffer moisture fluctuations, facilitate the drying process, and prevent oxidation.

Significant progress has been made in the research of microbial starters for fermented sausages, covering biochemical characteristics, physiological functions, antioxidant activity, safety evaluation, and volatile component analysis [9,12,13,14]. The long-term fermentation process driven by microbial communities through metabolic activities is a core factor determining product flavor, texture, and safety [15]. However, current research primarily focuses on the correlation between volatile flavor compounds and microorganisms, with a lack of systematic studies on the metabolic network analysis of microbial starters and non-volatile compounds (such as amino acids, organic acids, and nucleotides) in dry-fermented sausages. This limitation has led to an incomplete understanding of fermentation mechanisms and restricted technological development for optimizing product quality through precise control of microbial compositions. In the analysis of non-volatile compounds, high-performance liquid chromatography (HPLC), liquid chromatography–mass spectrometry (LC-MS), and amino acid analyzers have been widely used for quantitative detection of specific classes of metabolites. In recent years, nuclear magnetic resonance (NMR) technology has demonstrated unique advantages in low-molecular-weight metabolomics due to its non-destructive detection and high-throughput analysis of multiple metabolites [16,17,18]. This technology enables qualitative and quantitative analysis of multiple classes of substances, including free amino acids, organic acids, and nucleotides, through a single experiment and has been successfully applied to the metabolic profile analysis of dairy products [19], vegetable oils [20], and meat products [21,22,23]. For example, Yao et al. [24] used ^1^H NMR to analyze the characteristic flavor metabolites of river snails, while Zhou et al. [25] revealed that free amino acids, small peptides, and organic acids are the main metabolites contributing to the taste and flavor formation of modern processed hams. It is important to note that NMR technology serves as an auxiliary analytical tool in this study, combined with traditional chemical analysis methods, to systematically characterize the effects of different microbial inoculations on the distribution of non-volatile metabolites.

This study aims to investigate the impact of LAB as the initial culture on physicochemical properties and eating quality during the maturation process of dry-fermented sausages. Furthermore, this research will employ ^1^H NMR spectroscopy to identify non-volatile compounds in sausages and analyze the influence of LAB on the formation of these flavor-active compounds, thereby elucidating the role of LAB in the flavor development of sausages. Through this series of analyses, we anticipate gaining a comprehensive understanding of the specific effects of LAB on the quality characteristics of dry-fermented sausages.

## 2. Materials and Methods

### 2.1. Preparation of Lyophilized Fermenter

*Lactobacillus delbrueckii subsp. lactis* N102 and *Lactobacillus sakei H1-5* are strains that have been identified and preserved in the laboratory [26]. The strains were activated for three generations before being inoculated at a 1% volume into liquid culture, which was maintained until the end of the logarithmic growth phase. After centrifugation under sterile conditions (4 °C, 6000× *g*, 20 min), the supernatant was discarded, and the bacterial pellet was washed twice with sterile water. The pellet was then mixed with three times its volume of lyophilization protectant (11% skim milk powder, 8% sucrose, 4% monosodium glutamate, 3% glycerol) and homogenized for 10 min using a vortex mixer. The mixture was incubated at 37 °C for 30 min. After freezing at −80 °C for 12 h, the samples were lyophilized for 48 h in a freeze dryer and stored in sealed containers for subsequent use.

### 2.2. Preparation of the Dry-Fermented Sausages

Pork loins and back fats were chopped in a 4:1 ratio and blended with other ingredients, including 0.2% glucose, 0.3% sucrose, 0.3% black pepper, 0.3% white pepper, 0.1% garlic, 3.0% salt, and 0.05% sodium ascorbate. The compounded meat was divided into four groups for the fermentation of sausage, with the strains and inoculum levels of the starter cultures as shown in Table 1. The evenly mixed meat mixture was placed into a 36 mm collagen casing, with approximately 250 g filled per batch. After stuffing, the sausages were punctured with toothpicks to allow for air ventilation.

Subsequently, the sausages were placed in a fermentation chamber (Stagionello, Milano, Italy), with 15 sausages per group available for sampling during the fermentation process. Samples for analysis were taken at 0, 6, 13, 20, and 26 days into sausage fermentation. The specific parameter settings for the fermentation chamber are shown in Table 2.

### 2.3. Physical and Chemical Properties of Dry-Fermented Sausage

#### 2.3.1. Determination of pH

The pH was measured at 0, 6, 13, 20, and 26 days of fermentation using a pH meter (Mettler Toledo, Shanghai, China) in a homogenate sample made with 1:10 dilution using distilled water.

#### 2.3.2. Measurement of Water State

The water status was measured according to the method of Zhang et al. [27]. In detail, 5 g sausage sample is inserted in glass tube (2.5 cm diameter). The Carr–Purcell–Meiboom–Gill (CPMG) sequence was used to measure T_2_ and adjust the temperature at 32 °C. The proton resonance frequency adjusted to a starting frequency (SF) at 23 MHz and the offset frequency (O1) at 286.1954 kHz, with a cumulative number of scans (NS) of 6 and total data points (TD) of 384, 996, prg = 1, analog gain RG1 = 20, half echo time DL1 0.12 s, echo NECH = 1400, P1 = 17 mu s, P2 = 35 mu s, and Tw = 3500 ms. The water state measurement instrument low-field nuclear magnetic resonance (LF-NMR) (NIUMAG, SuZhou, China) was adjusted to magnetic resonance relaxation times of 0 to 10, 30 to 50, and 100–250 ms (millisecond) for hydration water, immobilized water, and free water, respectively [28].

#### 2.3.3. Thiobarbituric Acid Reactive Substance Measurement

The thiobarbituric acid-reactive substance (TBARS) of dry-fermented sausages was detected according to the method of Woldemariam [29]. In detail, a 3 g sausage sample was mixed with 10 mL of 7.5% trichloroacetic acid (TCA) and 50 µL of 7.2% butylated hydroxyanisole (BHA). To collect malondialdehyde (MDA) extract, the sample was homogenized for 2 min, centrifuged for 15 min at 1700× *g*, and then filtered using Whatman No. 4 filter paper. MDA was determined by reacting 1 mL of sample extract with 1 mL of 20 mM TBA in a screwcap tube. The tubes were heated at 90 °C for 30 min and allowed to cool down on ice. Finally, the absorbance was then measured at 532 nm with a spectrophotometer. The TBARS value was expressed as mg of malondialdehyde/100 g sausage.

#### 2.3.4. Determination of Nitrite Residue

The determination of residual nitrite content in fermented sausages complies with the national standard GB 5009.33-2016 [30] of the People’s Republic of China. The sample was extracted and purified by ion chromatography after precipitation of protein and removal of fat and then separated on an anion-exchange column with potassium hydroxide solution as eluent and detected by conductivity detector or ultraviolet detector. The analytes were characterized by retention time and quantified by external standard method.

#### 2.3.5. Determination of Biogenic Amine Content

Prepare the standard solutions and perform pre-column derivatization. Accurately weigh 50.00 mg of each amine (tyramine, putrescine, phenethylamine, cadaverine, tryptamine, histamine, spermine, and spermidine) and dilute to 50 mL with 0.4 mol/L perchloric acid solution. Prepare mixed standard solutions with final concentrations of 0.5, 1.0, 2.5, 5.0, 10, and 20 μg/mL by further dilution. To 1 mL of the mixed standard solution, add 200 μL of NaOH solution (2 mol/L) to adjust to alkaline conditions, followed by the addition of 300 μL of saturated NaHCO_3_ solution for buffering. Then, add 2 mL of dansyl chloride solution (10 mg/mL in acetone) and incubate in the dark at 40 °C for 45 min. After the reaction, add 100 μL of NH_4_OH to terminate the reaction, remove the excess dansyl chloride solution, and dilute to a final volume of 5 mL with acetonitrile. Filter the solution through a 0.22 μm filter membrane before analysis.

Sample Treatment: Add 20 mL of perchloric acid solution (0.4 mol/L) to 5 g of the sample, homogenize completely, and centrifuge at 4 °C and 4000× *g* for 10 min. Repeat the extraction with the pellet, and then take the supernatant, adjust the volume to 50 mL with 0.4 mol/L perchloric acid solution, and proceed with pre-column derivatization as described above for 1 mL of the sample solution.

Utilizing an LC-20AT high-performance liquid chromatography system (Shimadzu Corporation, Japan), the chromatographic conditions were established as follows: column: Shimadzu SB-C18; injection volume: 2 μL; column temperature: 30 °C; flow rate: 0.9 mL/min; ultraviolet (UV) detection wavelength: 254 nm; the elution program is detailed in Table 3.

### 2.4. Sensory Property Assessment of Dry-Fermented Sausage

#### 2.4.1. Texture Measurement

The casing of the sausage was removed, and each sausage was cut into cubic pieces with dimensions of 1 cm × 1 cm × 1 cm. Each group conducted three parallel experiments. A CT3 Texture Analyzer (AMETEK Brookfield, USA) was used, with the testing parameters set as follows: test speed of 1 mm/s; a 5 s interval between consecutive compressions; a compression rate of 50%; a P/35 probe; a load type of auto-5 g; and measurements were performed at room temperature.

#### 2.4.2. Color Measurement

Samples of the casing-removed sausage were sliced into 1 cm thick slices and collected from five distinct locations. Each group underwent three parallel experiments.

Measurement was performed using a CM-600d1 colorimeter (Konica Minolta, Tokyo, Japan). Before measurement, the device was calibrated using the integrated black and white boards. The L*, a*, and b* values were recorded.

#### 2.4.3. Sensory Evaluation

**Figure 1:**
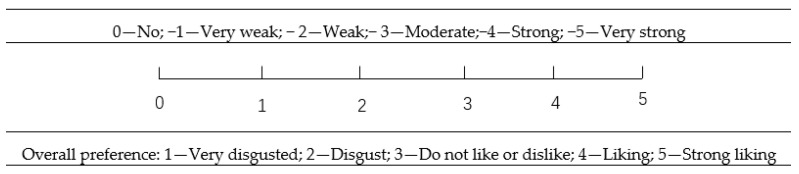
Sensory evaluation score chart.

Fifty individuals with a culinary expertise (25 males and 25 females) were recruited for a sensory assessment. The evaluation involved rating the sausages based on attributes such as redness, color uniformity, aroma, mouthfeel, sourness, and overall acceptability. A 5-point rating system was employed, as detailed in Figure 1. Additionally, assessors had the option to provide scores that straddled two scoring ranges.

### 2.5. NMR Measurement of Metabolite Differences Between Different Groups

#### 2.5.1. Sample Preparation

The purified cells were vortexed for 1 min in pre-cooled methanol (350 μL) and ultra-pure water (175 μL). Pre-cooled chloroform (350 μL) and ultra-pure water (350 μL) were added to each sample, followed by vortex mixing until homogeneous. The lower organic phase was collected by centrifugation at 1100× *g* for 15 min at 4 °C. This step was repeated four times, and the collected organic phases were combined and lyophilized for 24 h to obtain a freeze-dried powder of cell extracts. The freeze-dried powder of cell extracts was dissolved in 600 μL of phosphate buffer (NaH_2_PO_4_, K_2_HPO_4_, pH 7.4, 0.05% TSP and 100% D_2_O, 0.1 M). After vortex mixing and centrifugation at 1100× *g* for 15 min at 4 °C, the supernatant (550 μL) was transferred to a 5 mm NMR tube for instrumental analysis.

#### 2.5.2. NMR Spectroscopy

The ^1^H NMR experiments were conducted using an Avance III 600 MHz nuclear magnetic resonance spectrometer (Bruker, Berlin, Germany) equipped with a liquid helium cryogenic probe. The CPMG pulse sequence was utilized to identify small-molecule metabolites in each sample. Basic parameters were as follows: 90-degree pulse; width: approximately 8 μs; number of data points: 32 K; spectral width: 20 ppm; acquisition time: 1.36 s; number of scans: 512; number of dummy scans: 8; relaxation delay time: 2.3 s; temperature: 298 K.

#### 2.5.3. Data Analysis

To increase the signal-to-noise ratio, all 1D ^1^H NMR spectra were processed using MestReNova software (V7.0), which applied an exponential window function with a line widening factor of 1 Hz to the FID signals prior to Fourier transformation. After manual phase and baseline corrections, all samples’ NMR spectra were calibrated using the doublet in the α-glucose high-field region. The NMR spectra were integrated using appropriate software with the following parameters: integration range from 9.0 to 0.5 ppm, with an interval of 0.004 ppm, excluding the water peak (5.10–4.70 ppm) and methanol peak (3.38–3.31 ppm). The integrated data were normalized and subjected to multivariate data statistical analysis.

### 2.6. Statistical Analysis

Statistical analyses for the differences in pH, water state, TBARS, nitrite residue, texture, and color difference among groups were conducted with three experimental replicates, utilizing the General Linear Models procedure in SPSS Statistics 17 (Analytical Software, St. Paul, MN, USA). The data are presented as mean ± standard error. Analysis of variance (ANOVA) combined with Tukey’s multiple comparisons test was employed to assess the significance of the main effects (*p* < 0.05).

## 3. Results

### 3.1. Effects of Starter pH of Fermented Sausage

**Figure 2:**
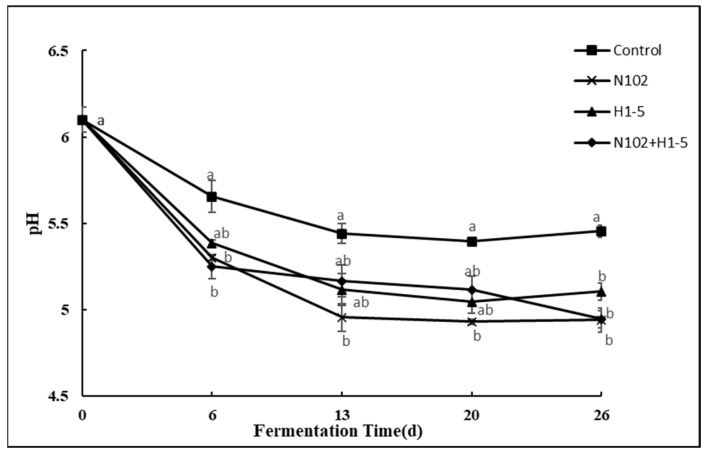
Evolution of pH value during the ripening of the different samples of sausage non-inoculated and inoculated with various starter cultures. Error bars refer to the standard deviations obtained from triplicate sample analysis. Different letters (a, b) indicate significant differences among different batches at the same fermentation time (*p* < 0.05). N102, *L. delbrueckii*; H1-5, *L. sakei*.

As shown in Figure 2, during the sausage fermentation process, the pH variation trends of all treatment groups were similar, with initial values all around 6.1. After 6 days, the pH of the control group dropped to 5.66 due to natural proliferation and acid production by LAB; however, the pH values of the experimental groups supplemented with starter cultures (N102, H1-5, and N105+H1-5 groups) were significantly lower (decreased to 5.30, 5.38, and 5.25, respectively). This difference can be primarily attributed to the directional metabolic activities of LAB in the starter cultures, and homofermentative strains efficiently produced lactic acid via the Embden–Meyerhof–Parnas (EMP) pathway, directly driving system acidification; heterofermentative strains simultaneously generated lactic acid and acetic acid through the phosphoketolase (PK) pathway. Notably, the combined inoculation group (N105+H1-5) exhibited the lowest pH (5.25), indicating a synergistic effect between homofermentative and heterofermentative strains—the combined acidification effect of lactic and acetic acids significantly enhanced the system acidity. This result confirms that the addition of exogenous fermentation agents plays a central role in the acidification process of sausages [31,32].

On the 26th day, the pH values of all groups except the N102+H1-5 group slightly recovered. This increase in pH could be attributed to the change in environment, which no longer favors the growth of LAB. Furthermore, ammonia from protein degradation may have partially offset the pH-lowering effect of LAB-produced acid. Compared to single inoculation, combined inoculation of LAB promotes organic acid production in sausages. Lower pH not only limits the growth of harmful bacteria and improves product safety, but it also enhances the texture and flavor of the sausage by regulating the proliferation of microorganisms and enzyme activity within it. Therefore, the addition of a fermentation agent is of great significance for the quality improvement of sausage.

### 3.2. Changes in Water State in Different Groups of Fermented Sausage

**Figure 3:**
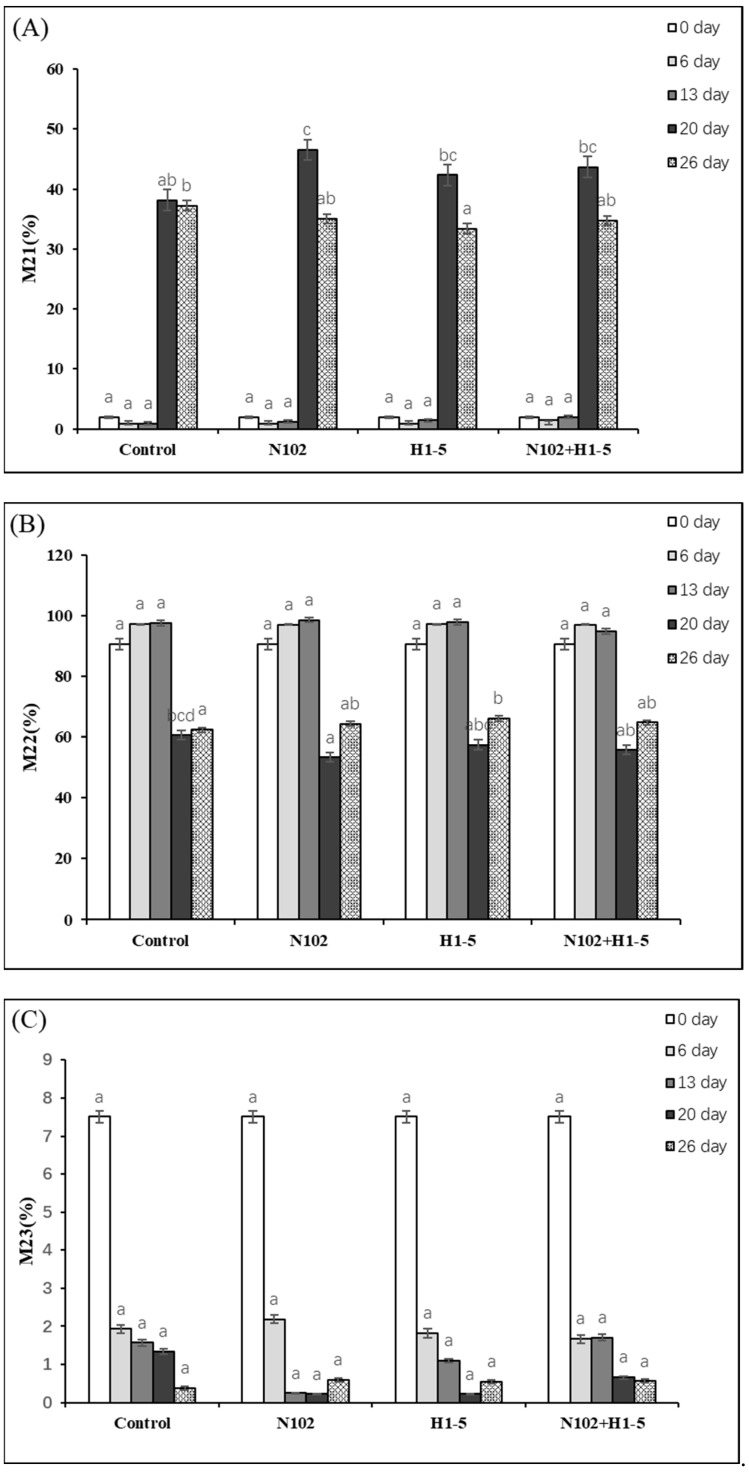
Water state of fermented sausage, where (**A**–**C**) indicate hydration water (M21), immobilized water (M22), and free water (M23), respectively. Error bars refer to the standard deviations obtained from triplicate sample analysis. Different letters (a–d) indicate significant differences among different batches at the same fermentation time (*p* < 0.05). N102, *L. delbrueckii*; H1-5, *L. sakei*.

The variations in different states of water (hydration water, immobilized water, and free water) in sausages exert significant impacts on microbial growth, as well as the juiciness, tenderness, and appearance of the products. Hydration water forms hydrogen bonds with proteins. Initially, the exposure of protein hydrophobic groups, triggered by a decrease in pH, leads to an increase in hydration water. Subsequently, throughout the day, its content fluctuates with protein cross-linking or degradation. As depicted in Figure 3A, on day 6, H1-5 exhibited the lowest percentage of minimum water of incorporation, at 0.84%, followed by sample N102 with 0.88%. By the end of fermentation, the percentage of hydration water in each group exceeded 33%. This phenomenon may be attributed to the structural changes in macromolecules such as proteins during the drying process or the action of emulsified salts in the sausages [33].

According to Figure 3B, the proportion of immobilized water showed an increasing trend until day 13, and a decrease on days 20 and 26 due to the drying process. Initially, the myofibrillar protein gel was densified, and the immobilized water increased to the peak value through the conversion of bound free water and immobilized water; later, the proportion decreased significantly to about 60% due to protein degradation and dehydration by drying. The amount of immobilized water retained was positively correlated with sausage tenderness, with the N102 group reaching a maximum value of 98.54% on day 13 and decreasing to a minimum of 53.22% on day 20. At the end of fermentation (day 26), the immobilized water retention was 64.73% in the N102+H1-5 group compared to 62.38% in the control group, indicating better stability of the myogenic fiber network.

As illustrated in Figure 3C, the percentage of free water decreased from an initial 7.51% to about 0.5% at the end of fermentation in all groups, including the control and N102+H1-5 groups, which decreased to 0.38% and 0.56%, respectively, on day 26. This change inhibited the growth of pathogenic microorganisms by reducing moisture activity, but excessive reduction may lead to dry and hard texture. Moisture migration was mainly regulated by drying dehydration in synergy with pH-driven protein denaturation, with the acidification process (pH 6.1 to 5.25) prompting protein aggregation at the isoelectric point and initially enhancing water-holding properties, while drying accelerated the evaporation of free water and the release of immobilized water. It is worth noting that the free water outside the myofibril network contains water in the sarcoplasmic proteins, and its reduction synergistically interacts with changes in pH and fat composition to improve sausage flavor and quality. The study by Moller [34] confirms that the fermentation process itself affects the water state transition and that pH, fat content, and other factors jointly regulate the dynamics of water distribution.

### 3.3. Effects of Starter on TBARS of Fermented Sausage

**Figure 4:**
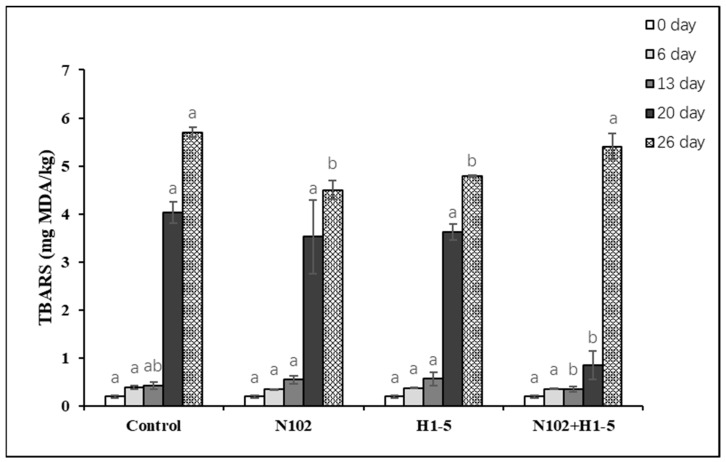
TBARS changes in dry-fermented sausage during fermentation. For thiobarbituric acid-reactive substances (mg MDA/kg meat), different letters (a, b) indicate significant differences among different batches at the same fermentation time (*p* < 0.05). N102, *L. delbrueckii*; H1-5, *L. sakei*.

The TBARS value was used to assess the secondary lipid oxidation of sausage by measuring the concentration of the secondary product malondialdehyde. As depicted in Figure 4, the TBARS values increased progressively with the drying and maturation of the sausage. During the initial 13 days, no significant differences in TBARS values were observed among the groups. However, on the 20th and 26th days, the TBARS values significantly increased, which may be attributed to the accelerated lipid decomposition during the sausage drying process, thereby enhancing the oxidation of unsaturated fatty acids. On the 20th day, the TBARS value of the N102 + H1-5 group was significantly lower than that of the control group (*p* < 0.05).

### 3.4. Changes in Nitrite Residue Content in Dry-Fermented Sausage

**Figure 5:**
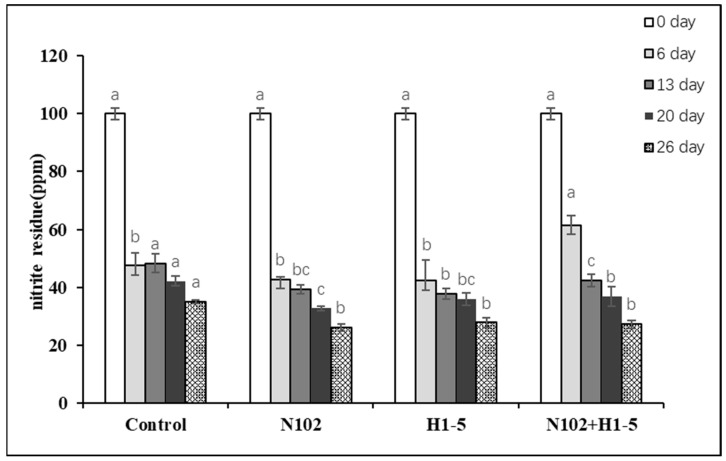
The nitrite residues in different dry-fermented sausages after production. Different letters (a, b, c) indicate significant differences among different batches at the same fermentation time (*p* < 0.05). N102, *L. delbrueckii*; H1-5, *L. sakei*.

As Figure 5 illustrated, nitrite levels in sausages from each group were monitored at multiple maturation stages. With an initial nitrite addition of 100 mg/kg, all groups exhibited elevated nitrite residues on day 6. From day 13 until the end of maturation, nitrite levels in all groups gradually declined. Notably, starter culture-inoculated groups showed significantly lower nitrite residues at each stage compared to the control group (*p* < 0.05).

In the final products, the N102, H1-5, and N102+H1-5 groups had nitrite residues below 30 ppm—a 70% reduction—whereas the control group exceeded this threshold. The mechanism involves LAB generating an acidic environment through lactic acid production, while their catalase activity promotes nitrite decomposition to nitric oxide (NO). This NO reacts with myoglobin to stabilize the sausage’s bright red color. Additionally, LAB facilitate nitrite conversion to ammonia, thereby inhibiting nitrosamine formation. Consequently, LAB not only reduce nitrite residues but also enhance color development and ensure product safety [35].

### 3.5. Analysis of Biogenic Amine Content in Dry-Fermented Sausages During Maturation

Biogenic amines at low concentrations participate in essential human physiological processes, including blood pressure regulation, thermoregulation, and gastric acid secretion. However, exceeding safety thresholds can lead to severe health risks, such as hypertensive crises and respiratory distress [36]. Among BAs, histamine is the most toxic. As shown in Table 4, histamine was undetected in all four sausage groups. Starter cultures had no impact on spermine levels, which originated from raw meat substrates. Putrescine concentrations remained low (<10 mg/kg) across all groups, well within safe limits.

Phenylethylamine, generated via phenylalanine decarboxylation, was detected at 61.51 mg/kg in the control group, compared to 22.73 mg/kg (N102) and 53.85 mg/kg (H1-5); other groups showed no detection. Tryptamine, produced by tryptophan decarboxylation, was absent in the H1-5 group, with experimental groups exhibiting significantly lower levels than the control (*p* < 0.05). Tyramine and cadaverine were present at higher concentrations in all groups. Tyramine, derived from tyrosine decarboxylation, had concentrations within the acceptable range (100–800 mg/kg), with the control group showing the highest levels, indicating that starter cultures inhibited tyramine production to varying degrees. Cadaverine, formed via lysine decarboxylation, along with tyramine and putrescine, is primarily produced by lactic acid bacteria, enterobacteria, and pseudomonads [37]. Total BA content in all groups remained below 1000 mg/kg (safe threshold), with starter-inoculated groups exhibiting lower totals than the control, demonstrating effective BA inhibition by the added cultures.

### 3.6. Texture Profile Analysis of Dry-Fermented Sausages During Maturation

**Figure 6:**
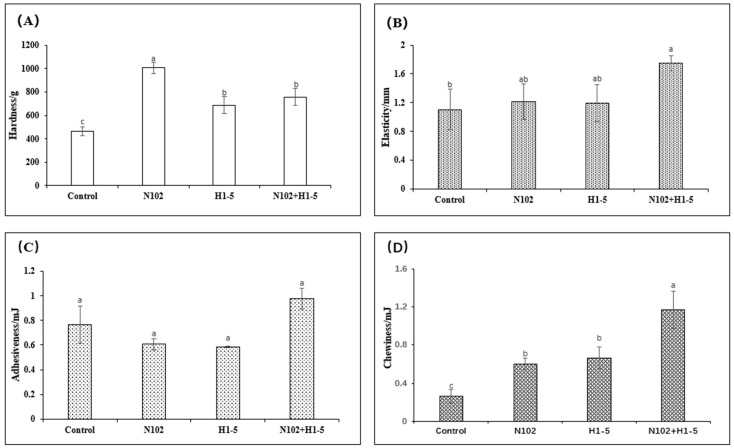
Texture analysis of fermented sausage by inoculating different starter cultures at maturity, where (**A**–**D**) indicate hardness (**A**), elasticity (**B**), adhesiveness (**C**), and chewiness (**D**), respectively. Different letters (a–c) indicate significant differences in values between different groups (*p* < 0.05). N102, *L. delbrueckii*; H1-5, *L. sakei*.

As depicted in Figure 6, the hardness, elasticity, adhesiveness, and chewiness of the sausages from four groups were measured after maturation. The texture of the sausages is influenced by various factors, such as pH levels and alterations in myofibrillar protein structure. The results indicate that there was no significant difference in adhesiveness among the sausage groups. The hardness of the experimental group was significantly higher than that of the control group (*p* < 0.05). The change in hardness may be attributed to the promotion of protein gelation as the pH decreases to the isoelectric point of the proteins, along with the gradual reduction in moisture content during drying. Compared to single-strain inoculation, mixed inoculation shows better qualities in terms of texture, with more moderate hardness and more elasticity of the flesh, resulting in a richer and more varied taste.

### 3.7. Analysis of Color Difference Changes in Dry-Fermented Sausages

**Figure 7:**
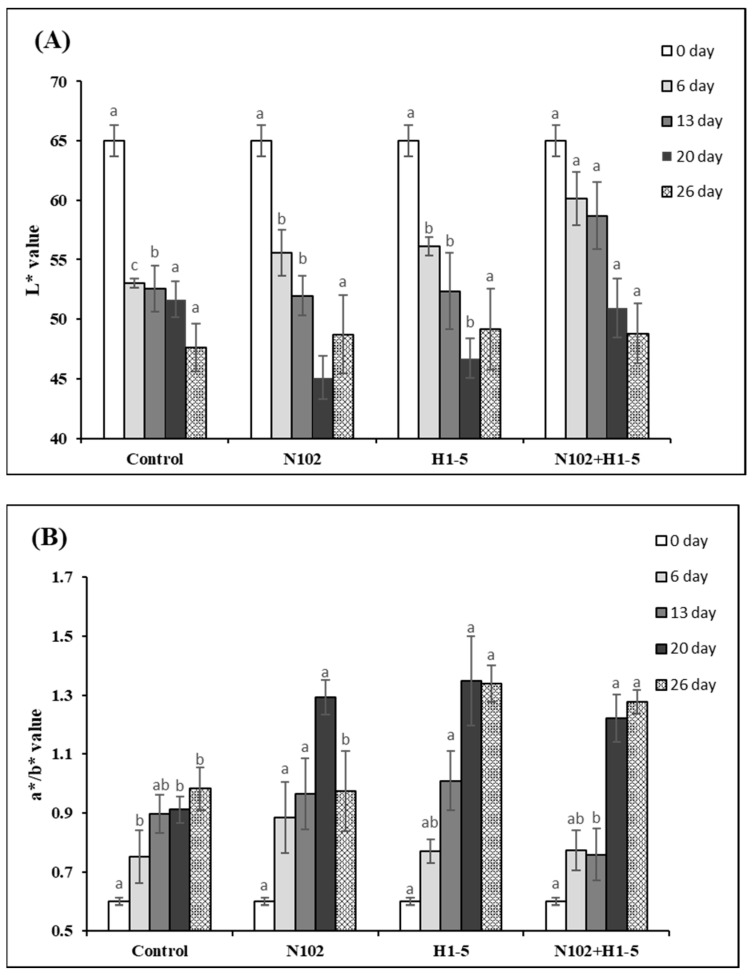
The color difference in dry-fermented sausages after production. (**A**) L* represents the brightness; (**B**) a* indicates the range from red to green, b* indicates the range from yellow to blue. The a*/b* ratio was used to assess color changes. Different letters indicate (a, b, c) significant differences in values between different groups at the same stage (*p* < 0.05). N102, *L. delbrueckii*; H1-5, *L. sakei*.

As shown in Figure 7, the L* values of all groups decreased with the drying and maturation of the sausage, which is attributed to the continuous reduction in moisture content, leading to a decrease in brightness values. The L* value of the experimental group on day 6 was significantly higher than that of the control group (*p* < 0.05). The a*/b* values of all groups showed a significant upward trend during the fermentation period and the early maturation stage, around 20 days, while the rate of increase slowed or some groups showed a downward trend in the late maturation stage. This may be due to the significant water loss in the fermented sausage during this period, which increases the concentration of myoglobin, thereby increasing the a* value. Additionally, under the action of microbial enzymes, sodium nitrite is converted to NO, which, when combined with myoglobin, forms nitroso myoglobin, a bright red pigment, thus increasing the a* value [38]. In the late maturation stage, the moisture content of the sausage becomes increasingly low, and the concentration of pigment substances leads to a slowing or decrease in the a* value [39]. The a*/b* value of the experimental group at the maturation stage, i.e., day 26, was significantly higher than that of the control group (*p* < 0.05). These results indicate that the addition of LAB fermentation agents can improve the color of the sausage, with a better effect observed in mixed fermentation with multiple strains.

### 3.8. Sensory Evaluation Results

**Figure 8:**
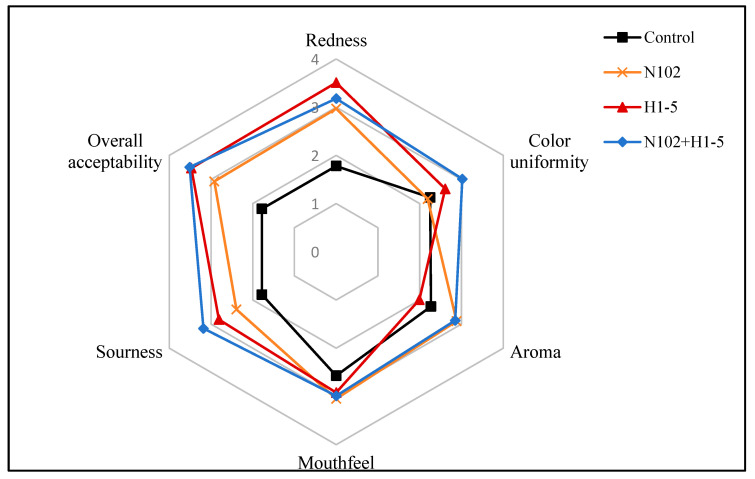
Sensory quality comparison of the different dry-fermented sausages.

In the sensory evaluation, sausages were scored for redness, color uniformity, aroma, mouthfeel, sourness, and overall acceptability. Previous analyses revealed divergent effects of different LAB starter cultures on sausage attributes. As shown in Figure 8, experimental groups achieved significantly higher color scores (redness and color uniformity) than the control group. Notably, the H1-5-inoculated sausages exhibited the highest redness scores, consistent with their elevated a*/b* values during ripening. The distinctive red-white marbling in sausage cross-sections, linked to processing steps like freezing and chopping, was evaluated for color uniformity.

Sausage aroma is a critical consumer preference factor. Data indicated that experimental groups generally received higher aroma scores than the control group, with the mixed starter culture group (N102+H1-5) outperforming both the control and single-strain groups. This suggests synergistic aroma enhancement by the mixed culture. In mouthfeel evaluations, experimental groups surpassed the control, aligning with their superior hardness, chewiness, and elasticity observed in textural analyses. The N102 group achieved the highest score, followed by the N102+H1-5 group, potentially attributed to its greater hardness.

Sourness correlated strongly with pH, with the control group’s lower score reflecting its higher pH. For overall acceptability, experimental groups outscored the control, with the N102+H1-5 group achieving the highest rating, indicating broad consumer preference. Collectively, these results demonstrate that LAB starter cultures—particularly mixed inoculations—significantly enhance sensory quality by improving color, aroma, mouthfeel, sourness, and overall acceptability, providing a robust strategy for sensory optimization in fermented sausages.

### 3.9. Differences in Metabolic Substances During the Maturation of Dry-Fermented Sausages

**Figure 9:**
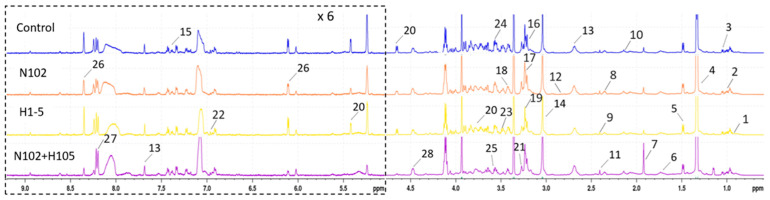
The 600 MHz ^1^H NMR spectra of serum obtained.

The maturation process of various sausage groups was investigated through the analysis of metabolites using NMR spectroscopy. The results are depicted in Figure 9 as ^1^H-NMR spectra, where the numbers denote the peak positions corresponding to individual metabolites. The spectral analysis revealed distinct differences among the different groups. After attribution of the metabolites in the spectra, the data are summarized in Table 5, with the numbers corresponding to those in Figure 9. A total of 28 substances were identified, including amino acids (isoleucine, leucine, valine, alanine, arginine, glutamate, glutamine, methionine, aspartate, taurine, glycine, phenylalanine); organic acids and their derivatives (succinate, lactate, acetate, creatine); peptides (anserine); lipids (glycerol, O-phosphocholine, sn-glycero-3-phosphocholine, trimethylamine N-oxide, 2-phosphoglycerate); carbohydrates (glucose); alcohols (myo-inositol); nucleosides (adenosine, inosine); and others (betaine, o-cresol). These non-volatile flavor precursors, including sugars, amino acids, creatine, peptides, and nucleotides, may influence the taste, flavor, tenderness, and juiciness of meat and meat products [40].

**Figure 10:**
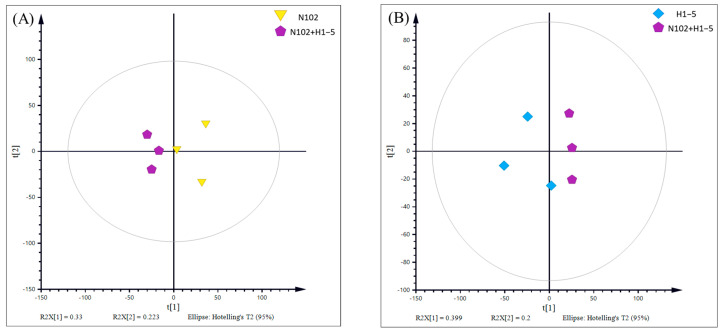
PCA score plots derived from ^1^H CPMG spectra from different contrast groups. (**A**) N102−N102 + H1-5; (**B**) H1-5− N102 + H1-5.

**Figure 11:**
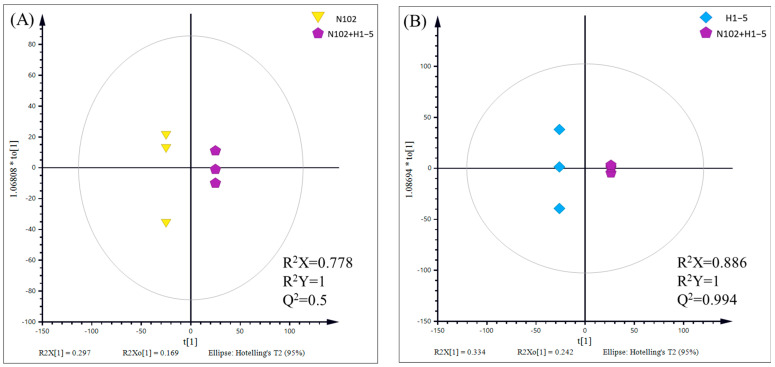
OPLS-DA score plot from different contrast groups. Q^2^ denotes the predictive power of the model. (**A**) N102−N102+H1-5, R^2^X = 0.778, R^2^Y = 1, Q^2^ = 0.5; (**B**) H1-5−N102+H1-5, R^2^X = 0.886, R^2^Y = 1, Q^2^ = 0.994.

To delve into the characteristic metabolic substances and their differences induced by the selected fermentation agents in dry-fermented sausage, a comparative analysis was conducted between two groups of samples (N102+H1-5-N102 and N102+H1-5-H1-5). The results of principal component analysis (PCA), as shown in Figure 10, reveal a clear trend of separation between these two groups of samples. This indicates that the metabolic profiles in the sausage have changed due to the different fermentation agents used, leading to significant metabolic differences between the groups. Furthermore, to further eliminate the impact of within-group variations and random errors on the analysis outcomes and to maximize the inter-group differences, orthogonal partial least squares discriminant analysis (OPLS-DA) was employed. As depicted in Figure 11, the OPLS-DA model exhibits a good performance in describing the samples, thereby providing further evidence of the significant metabolic disparities induced by different fermentation agents in the sausage.

The data in Table 6 present the significant differences in metabolite concentrations between the two groups along with their correlation coefficients. Upon comparing the fermentation outcomes of the mixed lactobacillus strain N102+H1-5 with those of *Lactococcus lactis* N102 and *Lactobacillus sakei* H1-5 when fermented individually, we observed a marked increase in the concentrations of various metabolites in the mixed fermentation group. Specifically, the contents of leucine, isoleucine, methionine, anserine, creatine, inositol, hypoxanthine, aspartate, glutamate, succinate, and acetate in the N102+H1-5 group were significantly higher than those in the groups fermented by N102 and H1-5 alone. These results indicate that the mixed fermentation approach effectively enhances the production of these specific metabolites.

During the fermentation of sausage, creatine serves as a primary organic acid and plays a pivotal role. Studies have shown that creatine is a key compound in muscle energy metabolism and performance, and it significantly contributes to enhancing the water-holding capacity of the product [41]. Consequently, the concentration of creatine may be a significant factor influencing moisture content changes in sausage during the fermentation process.

Carnosine and anserine are histidine dipeptides that are commonly found in the muscle tissue of most vertebrate species. They are categorized as carnosine-related compounds (CRCs), which are endogenous bioactive compounds renowned for their significant buffering capacity and antioxidant properties [42]. Additionally, carnosine is considered an additive factor for enhancing the nutritional quality of meat due to its anti-aging characteristics [43]. In the experimental group, the N102+H1-5 mixed strain fermentation group exhibited higher levels of carnosine and anserine compared to the single-strain fermentation groups, indicating that mixed lactic acid bacteria fermentation can more effectively enhance the antioxidant capacity of sausage. This enhancement may be a key factor contributing to the reduced rate of lipid oxidation in sausage.

Savoriness is a crucial component of meat flavor. The umami taste of meat primarily originates from nucleotides and umami amino acids (AAs). Branched-chain amino acids, such as leucine and isoleucine, play significant roles in the flavor development of sausage; IMP, GMP, and AMP are important components of umami in nucleotides [44]. Among these, IMP can impart umami, sweetness, and various flavors to foods with low concentrations [45]. Zhou et al. [46] classified free amino acids (FAA) into four categories based on flavor profiles, namely umami AAs, sweet AAs, bitter AAs, and tasteless AAs. The spectral data revealed that the majority of metabolites are related to umami taste. Glutamate and aspartate are categorized as umami AAs. IMP is an intermediate metabolite of four metabolic pathways, derived from the conversion of glutamate and aspartate. Taurine metabolism is related to IMP, which is primarily involved in glycine, creatine, and betaine. Glycine is classified as a sweet AA, providing sweetness and also enhancing the umami taste of food. Moreover, creatine and betaine affect the tenderness, water-holding capacity, and AA content of meat.

When comparing the N102+H1-5 mixed strain fermentation group with the single strain fermentation group, it was found that the AMP content was lower in the N102+H1-5 group, while the hypoxanthine content was higher than both groups. Glutamate and aspartate contributed significantly to the freshness of the meat products. The glucose content was also lower in the N102+H1-5 group, which may be due to the fact that the mixed fermentation accelerated the substrate utilization by the strains, which may have affected the IMP production and the freshness of the meat. This elevated metabolic activity may be a factor affecting the overall flavor and quality of the sausages.

## 4. Conclusions

In this study, the impact of various LAB inoculations on the physicochemical properties and eating quality of dry-fermented sausages during maturation was investigated. The maturation stages of the sausages were assessed at 0, 6, 16, 20, and 26 days by measuring physicochemical indicators and eating quality and by analyzing non-volatile components using ^1^H NMR spectroscopy to comprehensively understand the role of LAB in flavor development. In terms of safety, it was found that the rapid acid-producing metabolism of LAB (e.g., lactic acid fermentation) contributed to a significant decrease in sausage pH, and inoculation with LAB contributed to a rapid decrease in sausage pH, effectively inhibiting the growth of spoilage bacteria. In addition, the nitrite reductase produced by LAB metabolism accelerated NO_2−_ decomposition, and at the same time, through the metabolites (e.g., lactic acid, acetic acid) and nitrite forming a competitive reaction, it reduced the generation of nitrosamine precursors and accelerated the degradation of sodium nitrite. The LAB kept harmful biogenic amines, such as tyramine and histamine, below the detection limit by modulating amino acid decarboxylase gene expression, while enhanced monoamine oxidase activity further degraded potentially toxic amines. Inoculation significantly reduces the production of biogenic amines, thus ensuring that the product is safe for consumption. The introduction of lactic acid bacteria played a positive role in terms of edible quality. Specifically, the hybrid strain N102+H1-5 significantly slowed down the rate of MDA production and reduced lipid oxidation by secreting antioxidant enzymes such as superoxide dismutase (SOD) and glutathione peroxidase (GSH-Px).

Compared to the control group, the sausages in the experimental group exhibited better moisture content, texture properties (including hardness, chewiness, elasticity, and color), and received higher scores in sensory evaluations. Analysis with ^1^H NMR spectroscopy revealed that exogenous fermentations may have provided abundant substrates for amino acid metabolism, promoting the generation of umami amino acids. This process likely led to an increase in key flavor compounds such as methyl alcohols, aldehydes, and acids. Notably, the mixed fermentation with LAB strains N102 and H1-5 was more effective than single-strain fermentation in significantly promoting the formation of essential amino acids, flavor-related amino acids, and bioactive substances such as anserine and creatine, thereby enhancing the overall flavor and nutritional value of the sausages.

In summary, the inoculation of LAB is beneficial for improving the safety and eating quality of dry-fermented sausages, with the mixed inoculation of N102 and H1-5 showing more pronounced effects. Currently, the application of ^1^H-NMR technology for the analysis of sausage metabolites is in its developmental stage. Future research could further refine the metabolic profile of sausages by focusing on the pathway attribution of differential metabolites and the construction of metabolic networks. Additionally, it is necessary to explore the overall impact of the synergistic effects of LAB and other strains on the quality of dry-fermented sausages.

## Figures and Tables

**Table 1:** Strains and inoculations used for sausage fermentation.

Group	Strain	Inoculum
Control	Non-inoculation	---
N102	*L. delbrueckii subsp. lactis* N102	10^7^CFUg^−1^
H1-5	*L. sakei* H1-5	10^7^CFUg^−1^
H1-5+N102	*L. lactis subsp. lactis* N102+ *L. sakei* H1-5	10^7^CFUg^−1^ (1:1)

Note: control group: no inoculation of strains; experimental group N102: *Lactobacillus delbrueckii subsp. lactis* N102; H1-5: *Lactobacillus sakei* H1-*5*; H1-5+N102: *Lactobacillus delbrueckii subsp. lactis* N102 and *Lactobacillus sakei* H1-*5*; inoculated at 10^7^ CFU g^−1^ (1:1).

**Table 2:** Processing parameters of dry-fermented sausage.

Stage	Time (h)	Temperature (°C)	Humidity (%)	Stage	Time (h)	Temperature (°C)	Humidity (%)
1	22	22	80	5	18	17	71
2	20	20	65	6	21	15	73
3	18	19	67	7	21	14	76
4	22	18	69	8	Until maturity	11	37

**Table 3:** Gradient elution procedure.

Elution Time/min	Mobile Phase A/%	Mobile Phase B/%
0.0	35.0	65.0
5.0	30.0	70.0
20.0	0.0	100.0
24.0	0.0	100.0
25.0	35.0	65.0
30.0	35.0	65.0

Note: Mobile Phase A: water; B: acetonitrile.

**Table 4:** The contents of biogenic amines of dry-fermented sausages after maturation.

Group	Biogenic Amines (mg/kg)							
	Tryptamine	Phenylethylamine	Putrescine	Cadaverine	Histamine	Tyramine	Spermine	Total Amount
Control	44.28 ± 2.21	61.51 ± 3.08	0.71 ± 0.04	82.32 ± 4.12	-	473.34 ± 23.67	163.69 ± 8.18	825.85 ± 41.29
N102	32.83 ± 1.64	22.73 ± 1.14	4.26 ± 0.21	218.55 ± 10.93	-	397.02 ± 19.85	122.02 ± 6.10	797.41 ± 39.87
H1-5	-	53.85 ± 2.69	3.36 ± 0.17	79.29 ± 3.96	-	369.85 ± 18.49	135.34 ± 6.77	641.70 ± 32.09
N102+H1-5	27.72 ± 1.38	-	9.20 ± 0.46	137.63 ± 6.88	-	376.22 ± 18.81	152.18 ± 7.61	702.94 ± 35.15

Note: “-” represents “not detected”. N102, *L. delbrueckii*; H1-5, *L. sakei*.

**Table 5:** Peak matching assignment.

No.	Metabolites	No	Metabolites
1	Isoleucine	15	Phenylalanine
2	Leucine	16	O-Phosphocholine
3	Valine	17	sn-Glycero-3-phosphocholine
4	Lactate	18	Taurine
5	Alanine	19	Trimethylamine N-oxide
6	Arginine	20	Glucose
7	Acetate	21	Betaine
8	Glutamate	22	o-Cresol
9	Glutamine	23	myo-Inositol
10	Methionine	24	Glycine
11	Succinate	25	Glycerol
12	Aspartate	26	Adenosine
13	Anserine	27	Hypoxanthine
14	Creatine	28	2-Phosphoglycerate

**Table 6:** Metabolites with significant differences between groups.

Metabolites	(N102+H1-5)/N102		(N102+H1-5)/H1-5	
	Fold	VIP	Fold	VIP
Adenosine	0.52 *	1.68	0.38 *	1.56
Anserine	1.54 *	1.62	1.14 **	1.70
Hypoxanthine	1.37 *	1.61	1.70 *	1.59
Phenylalanine	1.29 *	1.61	0.77 *	1.65
o-Cresol	1.22 *	1.60	1.29 *	1.64
Glucose	0.40 *	1.73	0.35 *	1.66
2-Phosphoglycerate	1.13 **	1.77	1.14 *	1.53
Creatine	1.36 *	1.63	1.31 *	1.53
Betaine	0.80 *	1.60	0.38 *	1.56
Aspartate	1.16 *	1.65	0.46 *	1.62
sn-Glycero-3-phosphocholine	0.74 **	1.76	0.80 **	1.67
Glycerol	0.65 **	1.82	0.63 *	1.67
myo-Inositol	1.35 **	1.74	1.40 **	1.71
O-Phosphocholine	0.44 *	1.76	0.42 **	1.70
Glycine	0.65 **	1.82	0.65 *	1.65
Glutamate	1.20 *	1.75	1.12 *	1.48
Succinate	1.21 *	1.55	1.38 *	1.52
Methionine	1.20 *	1.75	1.36 *	1.54
Isoleucine	1.45 **	1.75	1.62 *	1.61
Arginine	4.17 **	1.79	4.16 *	1.67
Acetate	4.17 **	1.79	4.16 *	1.67
Leucine	1.21 *	1.62	1.15 *	1.41
Alanine	-	-	0.64 *	1.68
Valine	-	-	0.81 *	1.59
Taurine	-	-	0.66 *	1.65
Trimethylamine N-oxide	-	-	1.14 *	1.48
Glutamine	-	-	1.17 *	1.59

Note: The Fold value is the ratio of the mean integral area of the comparative group to the mean integral area of the control group. A Fold value > 1 indicates that the content of the substance corresponding to the chemical shift is higher in the comparative group than in the control group, and vice versa if it is lower; the higher VIP value indicates a more significant difference in the metabolite between the two groups. Generally, VIP > 1 suggests that the metabolite is a potential differentially abundant metabolite; the *p*-value was converted from the *t*-test; * indicates *p* < 0.05; ** indicates *p* < 0.01; (-) indicates that there is no significant difference in the substance between the two groups.

## Data Availability

The original contributions presented in the study are included in the article, further inquiries can be directed to the corresponding author.

## Abbreviations

Aw	Water activity
LAB	Lactic acid bacteria
CNS	Coagulase-negative staphylococci
HPLC	High-performance liquid chromatography
LC-MS	Liquid chromatography–mass spectrometry
NMR	Nuclear magnetic resonance
^1^H NMR	Proton nuclear magnetic resonance
CPMG	Carr–Purcell–Meiboom–Gill
SF	Starting frequency
NS	Number of scans
TD	Total data points
LF-NMR	Low-field nuclear magnetic resonance
TBARS	Thiobarbutric acid reactive substance
TCA	Trichloroacetic acid
BHA	Butylated hydroxyanisole
MDA	Malondialdehyde
UV	Ultraviolet
EMP	Embden–Meyerhof–Parnas
PK	Phosphoketolase
PCA	Principal component analysis
OPLS-DA	Orthogonal partial least squares discriminant analysis
CRCs	Carnosine-related compounds
AA	Amino acids
FAA	Free amino acids
SOD	Superoxide dismutase
GSH-Px	Glutathione peroxidase
